# A quasi-experimental study to determine the effects of a multifaceted educational intervention on hand hygiene compliance in a radiography unit

**DOI:** 10.1186/s13756-016-0133-4

**Published:** 2016-10-19

**Authors:** Margaret O’Donoghue, Suk-Hing Ng, Lorna KP Suen, Maureen Boost

**Affiliations:** The Squina International Centre for Infection Control, School of Nursing, The Hong Kong Polytechnic University, Hung Hom, Kowloon, Hong Kong

**Keywords:** Hand hygiene, Radiographer, Alcohol based hand rub, Compliance, Multifaceted intervention

## Abstract

**Background:**

Whilst numerous studies have investigated nurses’ compliance with hand hygiene and use of alcohol-based hand rub (ABHR), limited attention has been paid to these issues in allied health staff. Reports have linked infections to breaches in infection control in the radiography unit (RU). With advances in medical imaging, a higher proportion of patients come into contact with RU staff increasing the need for good hand hygiene compliance. This study aimed to evaluate effectiveness on compliance of an intervention to improve awareness of hand hygiene in the RU of a district hospital.

**Methods:**

A quasi-experimental study design including questionnaires assessing knowledge and attitudes of hand hygiene and direct observation of participants was used to evaluate an educational programme on hand hygiene of the RU of a large district hospital. All healthcare workers (HCW), comprising 76 radiographers, 17 nurses, and nine healthcare assistants (HCA), agreed to participate in the study. Of these, 85 completed the initial and 76 the post-test anonymous questionnaire. The hand hygiene compliance of all 102 HCW was observed over a 3-week period prior to and after the intervention. The 2-month intervention consisted of talks on hand hygiene and benefits of ABHR, provision of visual aids, wall-mounted ABHR dispensers, and personal bottles of ABHR.

**Results:**

Before the intervention, overall hand hygiene compliance was low (28.9 %). Post-intervention, compliance with hand hygiene increased to 51.4 %. This improvement was significant for radiographers and HCA. Additionally, knowledge and attitudes improved in particular, understanding that ABHR can largely replace handwashing and there is a need to perform hand hygiene after environmental contact. The increased use of ABHR allowed HCW to feel they had enough time to perform hand hygiene.

**Conclusions:**

The educational intervention led to increased awareness of hand hygiene opportunities and better acceptance of ABHR use. The reduced time needed to perform hand rubbing and improved access to dispensers resulted in fewer missed opportunities. Although radiographers and other allied HCW make frequent contact with patients, these may be mistakenly construed as irrelevant with respect to healthcare associated infections. Stronger emphasis on hand hygiene compliance of these staff may help reduce infection risk.

## Background

Hand hygiene is one of the most important measures in prevention of hospital-acquired infections [1]. However, healthcare workers’ (HCW) compliance with hand hygiene practice is frequently poor [2]. Interventions to improve hand hygiene have included provision of training courses for HCW on correct techniques and timing, and improved availability and encouragement of use of alcohol-based hand rubs (ABHR) in clinical areas. Several studies have shown that improved compliance could reduce incidence of healthcare-associated infections [3–5].

Many problems associated with traditional handwashing have been reduced by its replacement with ABHR [1]. However, successful decontamination can only be achieved if all sections of the hands come into contact with product requiring a sufficient volume to be utilized and spread over the hands. Thus, adequate training is essential. Since introduction of ABHR, numerous studies have confirmed its efficacy and cost effectiveness compared to traditional handwashing [6–8]. Many factors shown to adversely affect hand hygiene compliance, such as limited accessibility to handwashing facilities [9], insufficient time [10], and skin irritation [11] may be overcome by ABHR use. Various interventions have been attempted to enhance compliance including educational sessions [12] and performance feedback [13]. Educational interventions tend to have limited long-term effects on behavior, whereas performance feedback led to increased handwashing being sustained for some time after the intervention. However, it appears that pronounced and long-term improvements require an awareness intervention employing a multifaceted approach [14, 15]. In assessing compliance, direct observation provides a more accurate estimate [16, 17] than self-reports which generally overestimate compliance [18].

Since the introduction of ABHR, there have been several investigations of compliance performed on physicians, nurses and other ward personnel [8, 9], but much less attention has been paid to other HCW.

Radiology has a high potential for cross-infection as there is a need for close contact between the radiographer (medical imaging technologist) and the patient to ensure correct positioning. The emergence of new imaging modalities has led to radiological examination becoming an integral part of patient care [19]. Attendees at radiology units (RU) vary from severely ill inpatients to out-patients. However, contact between patients and RU staff, possibly leading to cross-infection, may be underestimated due to perception of time spent in the RU being brief or contact being perceived as low risk even though seemingly minimal contact can lead to contamination of HCWs’ hands [20]. During the SARS outbreak, three radiographers were infected while conducting X-rays of infected patients [21]. Although the nature of direct contact between RU staff and patients differs significantly from that between nurses and in-patients, and its duration is considerably shorter, there is a higher frequency of transient contact with a larger number of patients.

The impact of cross-infection in the RU is unknown, but there are reports of spread of healthcare-associated infections attributed to lapses in hand hygiene by radiographers [22, 23]. In addition, studies have shown contamination of radiography equipment associated with poor compliance with hand hygiene of the staff [24–27].

In most RU, ABHR is available and HCW participate in infection control programmes. However, there have been limited attempts to evaluate hand hygiene compliance of this group. It is possible that programmes designed for nurses are not suited to the pattern of activity in an RU.

This study aimed to determine knowledge, attitudes, and compliance with hand hygiene of HCW in an RU and evaluate effects of an educational intervention accompanied by increased availability of ABHR on these parameters.

## Methods

### Setting and participants

The study was performed in an RU, comprising 11 examination rooms, at a large district hospital serving a population of 1.1 million. The target population was HCW of the RU, comprising 76 radiographers, 17 nurses, and nine healthcare assistants (HCA). Radiologists were excluded as they have minimal patient contact. Each examination room had a handwashing sink and ABHR dispenser. There was no designated area for the dispenser placement and no staff member was designated to check and restock them.

### Sample size

Based on other studies [28, 29], a sample size of 200 potential hand hygiene opportunities and completion of questionnaires by 50 subjects was estimated to provide 80 % power to detect a 50 % change in compliance.

### Study design

**Fig. 1:**
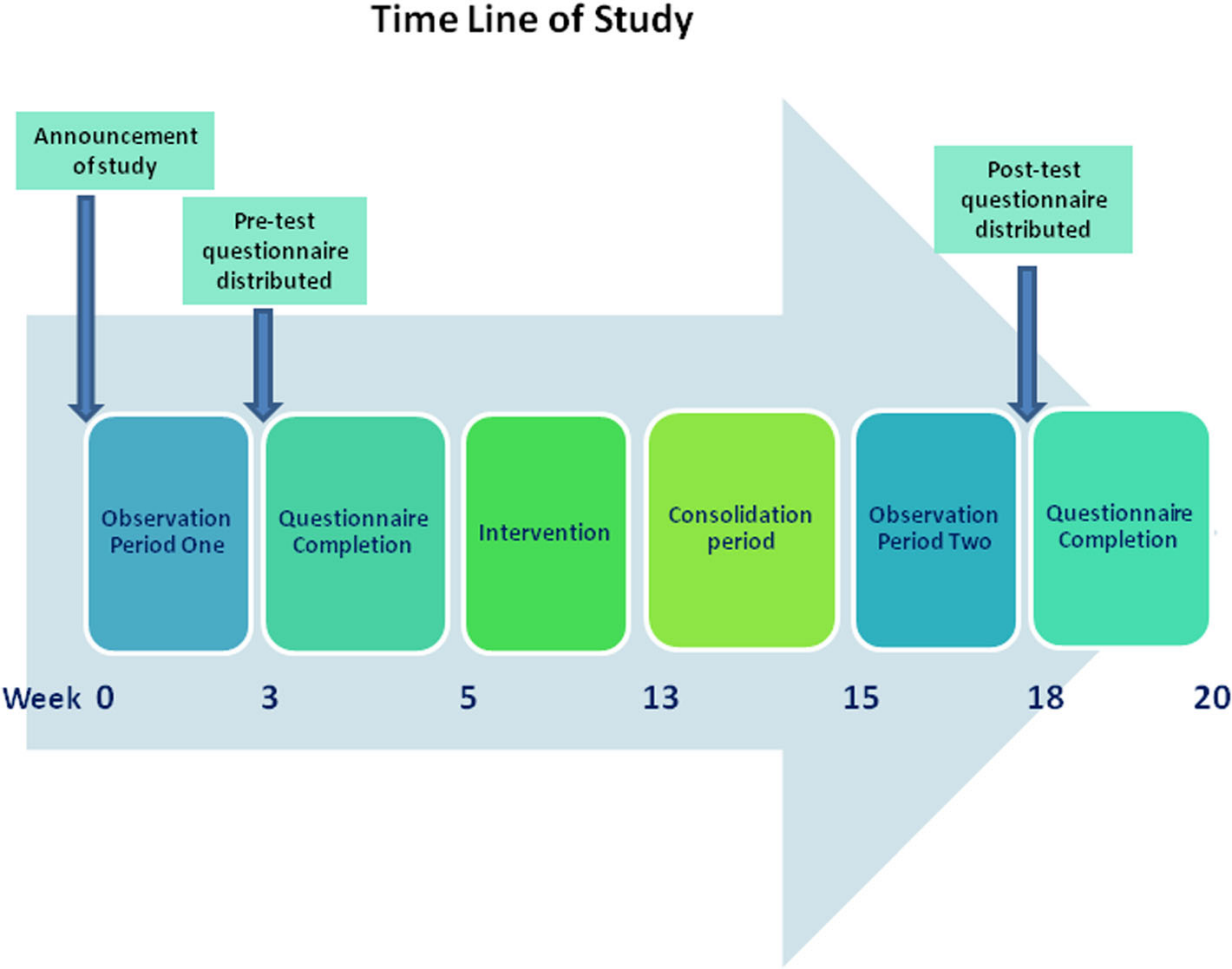
Timeline for implementation of an intervention to improve hand hygiene compliance in the RU

This was a quasi-experimental study consisting of a pre-test assessment, an intervention, and a post-test assessment. Subjects were observed for hand hygiene compliance and asked to complete a questionnaire to assess their knowledge and attitudes to hand hygiene before and after implementation of an intervention program. All HCW of the RU were invited to participate. Inclusion criteria were willingness to participate, full-time employment, opportunities for direct patient contact for a minimum of 50 % of working time, and no known allergy to ABHR. The study was conducted as shown in Fig. 1.

### Direct observation

Direct observation of compliance with hand hygiene was performed with the aid of a structured observational chart listing the five moments for hand hygiene based on WHO guidelines [16]. Hand hygiene after glove use was also observed. To prevent inter-observer variability, one trained observer, an experienced infection control nurse, recorded hand hygiene opportunities and classified actions as handwashing, ABHR use, or missed opportunity. The total number of patients seen and the number requiring special precautions were noted.

### Questionnaire

A self-report questionnaire using a five-point Likert scale was developed to assess knowledge of and attitudes to hand hygiene guidelines at baseline and again post-intervention. Demographic data was also collected.

The questionnaire’s content validity, as assessed by three infection control experts, was high (average congruence percentage rating of 93.3 %). The Chinese translation was independently back-translated into English and compared with the original. Reliability was determined by test-retest using 20 Accident & Emergency department nurses whose responses were compared on two occasions 2 weeks apart. Spearman’s rho coefficient was determined as 0.836 (*P* < 0.05).

### Subject recruitment and data collection

Two weeks before commencement of the study, all HCW were informed about the forthcoming observation and distribution of questionnaires. Observation, to determine baseline compliance with hand hygiene practice, comprised three randomly selected whole-day periods in three consecutive weeks (Fig. 1). The observation period, 9 am to 5 pm, covered the highest activity level and was sub-divided into sessions to cover the 11 examination-rooms. Lots were drawn each day to randomize the sequence in which rooms were observed.

To somewhat minimize the Hawthorne effect, the observer, a trained infection control nurse, was a staff member of the unit. HCWs were familiar with her presence and therefore, less conscious of her observation. The RD is generally busy and this also helped reduce awareness of observation. Observation was performed unobtrusively using a separate chart for each HCW. The exact date of each person’s observation was not announced.

Following completion of the observations, baseline questionnaires were distributed for return within 2 weeks to a drop box.

Post-test observations were conducted 2 weeks after completion of the intervention programme, followed by post-test questionnaire distribution. Pre- and post-test cohorts differed somewhat due to some staff changes, sickness and leave arrangements, but there was a high degree of overlap. Pre- and post-test assessments were analysed for changes in knowledge and attitudes, and hand hygiene compliance.

### Multifaceted intervention

During the 2-month intervention, all HCW received a pocket-sized ABHR and a pamphlet describing correct use. To facilitate hand hygiene, a further 20 ABHR dispensers were strategically placed beside each examination table, computed tomography examination bed, and radiographers’ work console of each room. The WHO formulation II (isopropyl alcohol 75 %, 1.45 % glycerol, 0.125 % hydrogen peroxide) was used. An HCA checked and restocked dispensers daily. Additional pocket-sized ABHR were freely available.

An awareness-raising campaign with an educational component aiming to encourage ABHR use and possibly increasing hand hygiene compliance was conducted before ABHR introduction. A 15-min refresher course covering the benefits of hand hygiene and correct use of ABHR was delivered three times at commencement and repeated 1 month later to ensure that the maximum number of staff were included. Pamphlets and A4 size posters were provided as reminders of correct ABHR technique.

### Data analysis

As exact pairing was not possible, the pre- and post-test data were regarded as independent. Pearson Chi-squared test was used to compare demographic characteristics of the groups. For the questionnaires, responses were assigned numerical values ranging from one for absence of knowledge or highly negative attitude to five for good knowledge and highly positive attitude. Median value and 25 and 75 % quartiles were determined.

Demographic characteristics were cross-tabulated against knowledge of indications for hand hygiene, attitudes to hand hygiene, and hand hygiene compliance rates based on observations. Compliance rates expressed as percentages were calculated as the proportion of actual hand hygiene actions performed over potential hand hygiene opportunities for the six indications. Ninety-five percent confidence intervals of independent proportions were calculated (http://vassarstats.net/prop2_ind.html). Compliance rates before and after were compared using Chi-squared test (Statistical Package for Social Science (SPSS) program version 20.0). The significance level was set at *p* < 0.05.

## Results

All 102 HCW indicated willingness to participate. Of these, 85 (84 %) completed both the baseline questionnaire and attended the briefing sessions and 74 (73 %) completed the post-test questionnaire (Table 1). Matching of questionnaires and observations was not possible as the questionnaires were anonymous. There was no significant difference in distribution of occupations, gender, or years of experience between the subjects completing the two questionnaires (Table 1) (*p* > 0.05).

**Table 1:** Comparison of subjects at baseline and post-intervention

		Pre-test	Post-test
Total number of questionnaires returned		85 (%)	74 (%)
Occupation	Radiographer	71 (84)	62 (84)
	Nurse	9 (11)	9 (12)
	HCA	5 (6)	3 (4)
Sex	M	47 (55)	40 (54)
	F	38 (45)	34 (46)
Years of experience	<1 year	7 (8)	3 (4)
	1–5 years	11 (13)	15 (18)
	>5 years	67 (79)	56 (76)

Numbers rounded to the nearest decimal place

*HCA* Healthcare assistant

At baseline, hand hygiene actions of 61 HCW were recorded. Sixty-two of 214 potential hand hygiene opportunities (either handwashing or hand-rubbing) were performed, representing 29 % overall compliance (Table 2). Patient turnover was high, with 83/214 (39 %) hand hygiene opportunities linked to new patients at baseline and 101/243 (42 %) post-test. Post-test compliance increased to 51 %, with 125 of 243 opportunities completed (Table 2). For both baseline and post-test, an average of seven potential opportunities was observed per subject. Approximately 60 % of opportunities involved before and after patient contact. Three instances of contact with body fluids or excretions or non-intact skin occurred during baseline observation but none post-test. No patients requiring special precautions were identified during the 6 days of observation (Table 2).

**Table 2:** Effects of an intervention programme on compliance with hand hygiene by occupation and by indications

		Pre-test hand hygiene performed (%)	Post-test hand hygiene performed (%)	Pre-test post-test difference % (95 % CI)	*p*
Total no. of hand hygiene opportunities (Overall Compliance %)		62/214 (29)	125/243 (51)	22 (14–31)	<0.01
Opportunities by discipline	Radiographer	35/132 (27)	79/154 (51)	25 (14–35)	<0.001
	Nurse	22/61 (36)	30/61 (49)	13 (4–30)	0.14
	HCA	5/21 (24)	16/28 (57)	33 (5–54)	0.01
Opportunities by Indications for hand hygiene	Before and after patient contact	28/126 (22)	62/145 (43)	21 (9–31)	<0.001
	After removing gloves	19/23 (83)	23/25 (92)	9 (−10–30)	0.33
	Before invasive procedures	5/12 (42)	5/12 (42)	0 (−35–35)	1.00
	After contact with inanimate objects in the immediate vicinity of the patient	8/50 (16)	35/61 (57)	41 (24–55)	<0.001
	After contact with body fluids or non- intact skin	2/3 (67)	0 (0)	NA	NA

HCW participation: Radiographers (baseline *N* = 41; post-test *N* = 51); Nurses (baseline *N* = 15; post-test *N* = 12); HCA (baseline *N* = 5; post-test *N* = 5)

*HCA* Healthcare assistant, *NA* Not applicable, *p* = Chi squared test

Both radiographers and HCA showed a significant improvement in hand hygiene compliance but nurses’ improvement did not reach significance (Table 2). Compliance was significantly increased for before and after patient contact and after contact with inanimate items (Table 2). Overall hand hygiene compliance increased significantly following the intervention by 23 % (*p* = 0.012). This 23 % improvement was entirely due to significantly increased use of ABHR (5 % before, 27 % after; *p* (Chi squared) = 0.003) as handwashing rates did not alter (24 % before and after). Missed opportunities reduced from 71 to 49 %.

Analysis of responses to the questionnaire indicated that initially respondents had quite good knowledge about hand hygiene as can be seen by generally high median scores (Table 3). As initial knowledge levels were good, there was little change for several of the questions (Q 1, 2, 5, 7). There was some improvement in understanding that hand hygiene is necessary before patient contact (Q6) and the need for rubbing until alcohol evaporated (Q10) but these improvements did not lead to a change in median score. The post-test median scores were improved for understanding that alcohol alone cannot be used for hand hygiene (Q3) but there was some confusion about appropriate use of ABHR as reflected in answers to Q8. Knowledge of MRSA transmission and the importance of environmental contamination improved considerably (Q4, Q9). This improved knowledge correlated well with less missed opportunities for these hand hygiene moments (Table 2).

**Table 3:**
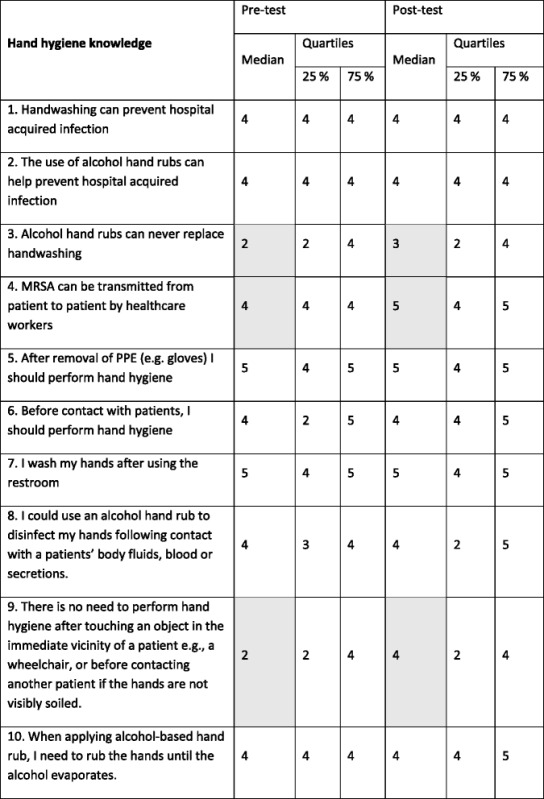
Comparison of pre-test and post-test scores for hand hygiene knowledge

For Questions 3, 8 and 9 the desired responses would be to disagree or strongly disagree and Likert scale scores were reversed for these questions. Shaded areas indicate a shift in median response

There was also an improvement in most median scores for attitude to hand hygiene following the intervention. Pre-intervention scores were high for questions relating to prevention of hospital-acquired infection indicating that most HCW already recognized the effectiveness of hand hygiene in preventing disease transmission. However, there was an increase in acceptance of ABHR (Q8). Appreciation of time saved by use of ABHR was also demonstrated by changes in responses to Q7 (Table 4).

**Table 4:**
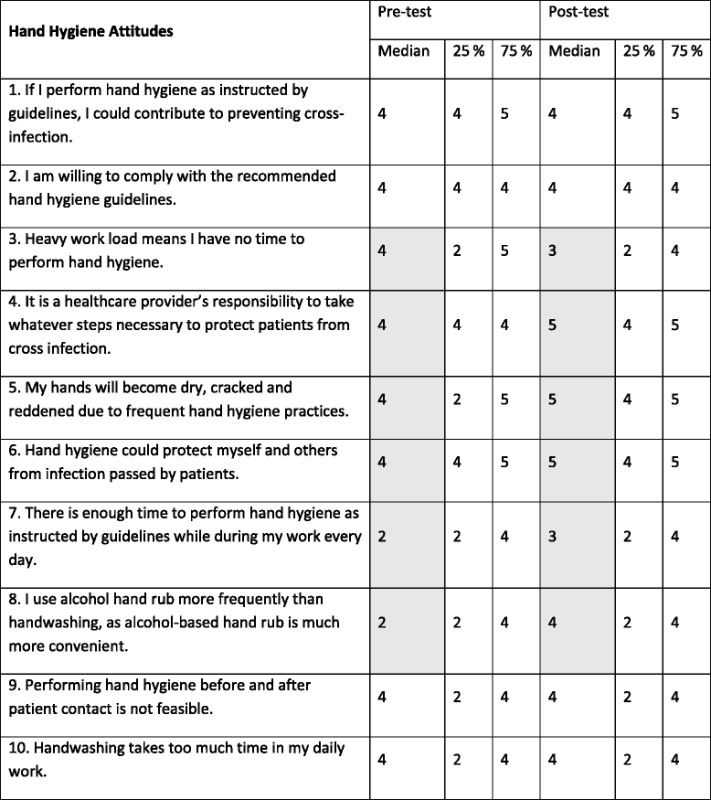
Comparison of hand hygiene attitudes pre and post intervention

Questions 1, 4 and 6 addressed prevention of hospital acquired infection, questions 2, 3, 7, 8, 9 and 10, compliance with recommended guidelines, and question 5, effects of alcohol on the skin

For Questions 3, 9 and 10 the desired responses would be to disagree or strongly disagree and Likert scale scores were reversed for these questions. Shaded areas indicate a shift in median response

## Discussion

This study showed that an awareness intervention with improved accessibility of ABHR led to a significant increase in hand hygiene compliance. This is one of the few reports of interventions for an RU and demonstrated that similar programmes to those used for ward staff can be successful elsewhere in the hospital. Participants were mainly radiographers but nurses and HCA in the unit were also involved. Whilst age distribution was fairly even, most staff had more than 5 years work experience indicating low staff turnover. Implementation of such programmes may be more cost effective if staff turnover is low.

Pre-intervention compliance was low overall being similar in radiographers and HCA but approximately 10 % higher for nurses. This agrees with earlier reports which noted compliance of nurses was highest of the medical personnel studied [29, 30]. Following the intervention, overall hand hygiene improved significantly. These findings were similar to those of previous studies in which post-intervention compliance rates of HCW of approximately 50 % were reported [31]. The improvement was greatest for HCA. A previous study showed that these employees are particularly receptive to hand hygiene education [32] which may be linked to lower pre-intervention knowledge.

Although the rate of nurses’ compliance increased, this did not reach statistical significance possibly due to both the initially higher baseline level of compliance and relatively low number of nurses employed in the RU. In addition, the attendance rate of nurses at the educational briefing was considerably lower (53 %) than that of radiographers (93 %). The attendance rate of the HCA was also low (53 %) but for workers with lower educational achievement levels, visual stimuli such as posters and changes in practice by peers and other staff present have been shown to be effective [32]. However, in spite of significant improvement, almost half of hand hygiene opportunities were still missed indicating that further reinforcement of education may be needed. Other studies performed with nurses have demonstrated that repeated interventions are required to achieve high levels of compliance [4]. In addition, for sustained improvement in hand hygiene behaviour, regular feedback to HCW may be needed [4, 33].

There were few missed opportunities for hand hygiene after removing gloves (17 %) and after the intervention the slight improvement did not reach statistical significance. Hand hygiene is a complex behaviour which includes an inherent component that is a natural self-protecting response to visibly or conceptually contaminated hands and acts as the principal driver for hand hygiene. In contrast, adopting practices of hand hygiene which are not instinctive, the so-called elective component, usually needs to be reinforced. Many hand hygiene opportunities in the hospital would not be considered as potential threats in the community and thus fail to trigger an intrinsic action [34]. This may help explain the poor pre-intervention compliance with hand hygiene after contact with inanimate objects. As would be expected in the RU, most hand hygiene opportunities were before and after patient contact but these moments were missed on 78 % of occasions. Without appropriate reinforcement of the importance of the elective component of hand hygiene, such opportunities would easily be neglected. Following the intervention, there was significant improvement in hand hygiene both before and after patient contact and after contact with inanimate objects. This agrees with the behavioural explanation that to improve hand hygiene compliance, the elective component needs to be influenced [34]. Formulating an effective interventional program for the RU presents a considerable challenge as there is a need to enhance frequent elective hand hygiene practices.

Significant improvement in hand hygiene compliance was almost entirely attributable to improved utilization of ABHR, with little change in hand-washing. This concurs with other reports on interventions involving acceptability of ABHR [15, 35]. Interestingly, there was no change in uptake of opportunities for hand hygiene before invasive procedures. Numbers of such invasive procedures were low with only 12 in both the pre-test and post-test periods and were performed by a limited number of staff. Nevertheless, more attention needs to be paid to compliance with this moment.

Direct observation provides a more accurate assessment of hand hygiene than self-report [36]. However, the Hawthorne effect must always be considered and minimized. In this study, the observer made every effort to perform the observations unobtrusively. In addition, to further minimize the Hawthorne effect, written consent of the staff was not sought to avoid arousing attention towards the observation process. Nevertheless, it is recognised that there was potential for Hawthorne effect in both sets of observations but it is likely that this effect was similar in both observation periods. Ideally, staff should perform their routine duties according to their usual practice, with the only influencing factors being the effects produced by the intervention. A similar approach has been adopted in other studies [15, 29]. It has been concluded that it is ethical to conduct studies involving risk-free practices such as hand hygiene without informed consent [37]. However, anonymity and privacy of the staff were strictly respected and staff were aware that a study was in progress.

Baseline assessment showed that hand hygiene knowledge and attitudes was acceptable and, notably, staff believed that compliance with recommended guidelines would have a positive outcome in terms of reducing hospital-acquired infection. The intervention led to improvements, in particular, staff indicated that they had enough time to comply with recommended guidelines. This may largely be attributable to acceptance of ABHR use as most staff were previously unaware that ABHR could replace handwashing for many hand hygiene moments. However, the increased diversity of responses to Q8 in the knowledge section did indicate there was some confusion about when it was inappropriate to use ABHR and the need for hand washing in cases of heavy soiling should be better explained in an intervention. Overall ABHR acceptance and observed use was supported by self-reported more frequent use after the intervention. It has previously been reported that staff have greatest difficulty with hand hygiene compliance during busy periods or when they feel overwhelmed [20, 38].

Similar improvement in knowledge was noted for the need for hand hygiene for other moments. Other studies have demonstrated that improved knowledge correlates with increased compliance with hand hygiene guidelines [39].

The questionnaires were designed particularly for the working environment of the RU and differed from those used in ward settings where staff are usually more aware of cross-infection. Analysis revealed there was considerable need for improvement of knowledge and attitudes with respect to ABHR, confirming the requirement for improved education for these workers. Specifically, as recommended by the WHO [16], hand hygiene intervention programmes should be tailored to match the cultural and environmental setting and the infection risk of frequent and brief contacts with patients should be clearly conveyed.

There were some limitations to this study. In contrast to other studies which have used indirect product use as an indicator of compliance rather than direct observation [37], we did not estimate amounts of ABHR consumed. However, product usage may not indicate appropriate use and other factors such as different staff hand hygiene practice, use by patients and families and other variables may affect this parameter [40].

Most staff participated in the study but a small number some could not be involved, due to leave arrangements and shift duties and or not attending the briefing. The baseline -post-test study design did not involve a control group, but this approach is considered acceptable for hand hygiene studies [37].

Numbers of nurses and HCA were low as is usual in an RD unit and so, improvements in these groups of workers may not be as accurate as for radiographers especially as the attendance of the former groups at the briefings was lower than that of the radiographers. For both groups, only half received the educational intervention and so, improvements observed may not reflect its effects.

The effect of the 2-month intervention was evaluated 2 weeks after its completion. It could be argued that a longer intervention could produce greater effects but there is also a risk that prolonging a programme may lead to participant fatigue accompanied by diminishing success. It would be useful to re-assess the effect after a longer period to determine if the increased compliance was sustainable. However, most studies on other healthcare workers have demonstrated that re-enforcement of training is needed to maintain or improve hand hygiene compliance [16].

## Conclusions

Observation confirmed the high frequency of short term patient contact in the RU, suggesting such settings are particularly suited to the use of ABHR for hand hygiene. The intervention was effective in enhancing the hand hygiene practice of the staff and led to good acceptance of ABHR which may reduce infection risk. However, although compliance increased significantly after the intervention, only half of hand hygiene opportunities were performed suggesting the need for repeated interventions. Regular scheduled refresher courses should be provided in order to sustain hand hygiene compliance.

## Abbreviations

ABHR: Alcohol-based hand rub
HCA: Healthcare assistant
HCW: Healthcare workers
RU: Radiography unit
WHO: World Health Organization
